# Maternal Serum, Cord and Human Milk Levels of Per- and Polyfluoroalkyl Substances (PFAS), Association with Predictors and Effect on Newborn Anthropometry

**DOI:** 10.3390/toxics11050455

**Published:** 2023-05-14

**Authors:** Maya Mahfouz, Mireille Harmouche-Karaki, Joseph Matta, Yara Mahfouz, Pascale Salameh, Hassan Younes, Khalil Helou, Ramzi Finan, Georges Abi-Tayeh, Mohamad Meslimani, Ghada Moussa, Nada Chahrour, Camille Osseiran, Farouk Skaiki, Jean-François Narbonne

**Affiliations:** 1 Department of Nutrition, Faculty of Pharmacy, Saint Joseph University of Beirut, Medical Sciences Campus, Damascus Road, P.O. Box 115076, Riad Solh Beirut 1107 2180, Lebanon; mireille.harmouche@usj.edu.lb (M.H.-K.); yara.mahfouz@net.usj.edu.lb (Y.M.); khalil.helou@usj.edu.lb (K.H.); 2 Industrial Research Institute, Lebanese University Campus, Hadath Baabda P.O. Box 112806, Lebanon; chem@iri.org.lb; 3 School of Medicine, Lebanese American University, Byblos 1102 2801, Lebanon; psalameh@ul.edu.lb; 4 Institut Polytechnique UniLaSalle, Collège Santé, Equipe PANASH, Membre de l’ULR 7519, Université d’Artois, 19 Rue Pierre Waguet, 60026 Beauvais, France; hassan.younes@unilasalle.fr; 5 Hotel-Dieu de France, Saint Joseph University of Beirut Hospital, Blvd Alfred Naccache, Beirut P.O. Box 166830, Lebanon; ramzifinan@hotmail.com (R.F.); georges.abitayeh@gmail.com (G.A.-T.); 6 General Management, Chtoura Hospital, Beqaa, Lebanon; meslimanimhd@gmail.com; 7 Department of Obstetrics and Gynecology, Chtoura Hospital, Beqaa, Lebanon; ghada.a.moussa@gmail.com; 8 Department of Obstetrics and Gynecology, SRH University Hospital, Nabatieh, Lebanon; nadachahrour7@gmail.com; 9 Department of Obstetrics and Gynecology, Kassab Hospital, Saida, Lebanon; camilleosseiran@hotmail.com; 10 Department of Molecular Biology, General Management, Al Karim Medical Laboratories, Saida, Lebanon; farouk.skaiki@st.ul.edu.lb; 11 Laboratoire de Physico-Toxico Chimie des Systèmes Naturels, University of Bordeaux, CEDEX, 33405 Talence France; narbonne.jf@gmail.com

**Keywords:** human biomonitoring, persistent organic pollutants, cord, human milk, newborn, perinatal exposure, pregnant women, exposure predictors

## Abstract

Background: The understanding of per- and polyfluoroalkyl substances (PFAS) health effects is rapidly advancing among critical populations. Therefore, the objective of this study was to assess PFAS serum levels among Lebanese pregnant women, cord serum and human milk levels, their determinants, and effects on newborn anthropometry. Methods: We measured concentrations of six PFAS (PFHpA, PFOA, PFHxS, PFOS, PFNA and PFDA) using liquid chromatography MS/MS for 419 participants, of which 269 had sociodemographic, anthropometric, environmental and dietary information. Results: The percentage of detection for PFHpA, PFOA, PFHxS and PFOS was 36.3–37.7%. PFOA and PFOS levels (95th percentile) were higher than HBM-I and HBM-II values. While PFAS were not detected in cord serum, five compounds were detected in human milk. Multivariate regression showed that fish/shellfish consumption, vicinity to illegal incineration and higher educational level were associated with an almost twice higher risk of elevated PFHpA, PFOA, PFHxS and PFOS serum levels. Higher PFAS levels in human milk were observed with higher eggs and dairy products consumption, in addition to tap water (preliminary findings). Higher PFHpA was significantly associated with lower newborn weight-for-length Z-score at birth. Conclusions: Findings establish the need for further studies, and urgent action to reduce exposure among subgroups with higher PFAS levels.

## 1. Introduction

Per- and polyfluoroalkyl substances (PFAS), also known as “forever chemicals” [1], are anthropogenic chemical compounds that are not naturally present in the environment; due to their physicochemical properties, they have been used in industry and in products intended for human consumption since the 1950s [2,3]. The main source of exposure to PFAS for the general population is through ingestion, including notably contaminated drinking water [4]. Regarding the dietary exposure to PFAS, the main food sources are fish and seafood; red meat; chicken; eggs and egg products; and dairy products [5,6,7,8]. 

While perfluorooctane sulfonic acid (PFOS), perfluorooctanoic acid (PFOA), perfluorohexane sulfonate (PFHXS) and their salts and related compounds were listed among the new persistent organic pollutants (POPs) under the Stockholm Convention, the listing of long-chain perfluorocarboxylic acids (LC-PFCAs) is being reviewed to be added [9]. Although efforts have been made to reduce the use of these chemicals, the understanding of their adverse health effects is still rapidly advancing [10], with special attention to critical populations. In fact, numerous studies have been conducted to assess levels of PFAS among pregnant women [11,12,13,14,15,16,17,18], with studies showing higher risks of hypertension, pre-eclampsia, and gestational diabetes [10,19,20]. PFAS can reach the fetus through transplacental transfer, although the transfer mechanism is still inconclusive [21]. PFAS can also reach the newborn through human milk, although breastfeeding’s benefits outweigh the downside of such a risk [22,23]. PFAS exposure during pregnancy or through human milk has been associated with increased risk of lower birth weight and neurodevelopmental problems, as well as a decreased immune response to vaccines in children [10,19,20]. 

In Lebanon, the “National Implementation Plan on Persistent Organic Pollutants” was issued in 2017 to examine POPs considered newly dangerous in the Stockholm Convention [24]. This document reported that in Lebanon, the main use of PFOS took place between 1970 and 2002, in particular through the use of fire extinguishing foams containing PFOS, following fires in residential, commercial and industrial areas. Consequently, there is potentially groundwater contamination at several sites [24]. Moreover, recent findings from Lebanon showed the detection of PFOA and PFOS in human milk samples [25]. 

Thus, the objective of the current study was to assess the serum levels of PFAS in a sample of Lebanese pregnant women, their levels in cord serum and human milk, their dietary, environmental, and sociodemographic correlates, as well as their effect on newborn anthropometry.

## 2. Materials and Methods

### 2.1. Study Design 

The study was conducted in Lebanon between March 2018 and January 2021. In total, 419 pregnant women without diseases and residing in Lebanon for the last 10 years were recruited at delivery at hospitals in Beirut and Beqaa governorates, and at gynecologists’ clinics in South and in Nabatieh. Upon recruitment (around delivery), maternal blood samples were collected (in vacuum tubes (BD Vacutainer, Plymouth, UK) without anticoagulants and centrifuged to extract serum at 3500 rpm for 15 min), and questionnaires were administered. Out of 419 participants, 269 had complete data regarding sociodemographic, environmental, anthropometric and dietary variables. Sociodemographic variables included age, crowding index and education. Data was also collected about parity, having been breastfed, smoking, and alcohol consumption. The questionnaires also included environmental questions about vicinity to illegal incinerations, geographical vicinity to landfills, and geographical vicinity to factories. Maternal anthropometric measurements included height, pre-pregnancy weight (from which BMI in kg/m^2^ was calculated), pre-pregnancy and pregnancy weight loss from restrictive diet, and gestational weight gain. The Institute of Medicine (IOM) guidelines were used to categorize gestational weight gain (GWG) [26]. During that same face-to-face interview, food consumption frequencies (in portions per week) of food groups deemed potential sources of PFAS were collected, covering the period prior to pregnancy. These food groups were fish and shellfish, red meat, poultry, dairy products, eggs and fruits [27]. More details are available elsewhere [28]. 

Cord blood samples were collected during delivery for 59 participants in order to be tested for pollutants. After delivery, a subsample of 41 women agreed and were able to provide a human milk sample. Human milk sampling was performed at the participants’ homes, between 3 and 8 weeks postpartum, based on the “Guidelines for Organization, Sampling and Analysis of Human Milk on Persistent Organic Pollutants” [29]. Next, 30 mL were collected by hand expression into a sterilized glass jar. The sample was stored in the refrigerator at 4 degrees for no more than 3 days if needed and was eventually stored at −20 degrees until analysis. During the milk collection phase, follow-up questions were added including use of tap water.

The chemical analyses were performed at the accredited Lebanese reference laboratories “Industrial Research Institute” (IRI)—Eurolab Lebanon (accredited by ANAB, USA, for ISO 17025 (testing and calibration) and ISO 17020 (inspection) and member of the International Accreditation Forum (IAF) and International Laboratory Accreditation Cooperation (ILAC), European federation of National Associations of Measurements, testing and Analytical Laboratories (EUROLAB)). 

All participants signed an informed consent prior to participation. This work was approved by the ethics committee of Saint Joseph University of Beirut (CEHDF 969), in addition to the collaborating hospitals and gynecologists. All procedures performed were in accordance with the 1964 Helsinki declaration and its later amendments or comparable ethical standards.

### 2.2. Toxicant Analysis in Serum and Milk

The following PFAS were quantified: perfluorohexanesulphonic acid (PFHXS), perfluoroheptanoic acid (PFHpA), perfluorooctanoic acid (PFOA), perfluorononanoic acid (PFNA), perfluorodecanoic acid (PFDA) and perflucorooctane sulfonic acid (PFOS). These specific PFAS congeners were quantified due to their toxicological importance, as stated by the Stockholm Convention [9] and major toxicological entities, namely the “Agency for Toxic Substances and Disease Registry” (ATSDR) [4] and the “Environmental Protection Agency” (EPA) [30]. These congeners are long-chain PFAS known for being extremely persistent and toxic, as well as for their bioaccumulation potential [31]. Key entities have been working towards the phase out of their production and use [32]. They are widely tested in international studies, and have been associated with a number of health effects, namely increases in serum lipids and pregnancy-induced hypertension and/or pre-eclampsia [4].

#### 2.2.1. Principle

To quantitatively detect PFASs, we used an online solid phase extraction coupled to high performance liquid chromatography-turbo ion spray ionization-tandem mass spectrometry (online SPE-HPLC-TIS-MS/MS).

After dilution with formic acid, we injected one aliquot of 50 μL of serum (and, accordingly, 50 μL of human milk) into a commercial column switching system, allowing for the concentration of the analytes on a solid-phase extraction column. Separation of the analytes from each other and from other serum components (accordingly milk components) was achieved with high-performance liquid chromatography. Detection and quantification were done using negative-ion electrospray ionization and tandem mass spectrometry. This method allowed for rapid detection of these PFASs in human serum and milk with limits of detection in the low parts per billion (ppb or ng/mL) range.

#### 2.2.2. Reagents, Solutions and Materials 

Methanol (MeOH), acetonitrile and water were HPLC-grade and purchased from Fischer Scientific. Formic acid (99%) was purchased from Merck. Acetic acid (glacial) was purchased from VWR International. PFASs were purchased form Wellington Laboratories (Guelph, ON, Canada). HyperSep C8-SE (7 µM) cartridge were purchased form Thermo Fisher Scientific. Further materials used included the following: Chromolith^®^ HighResolution RP-18e column (4.6 × 100 mm) (Merck KGaA, Darmstadt, Germany); Chromolith^®^ HighResolution RP-18e guard column (5 × 4.6 mm) (Merck KGaA, Germany); Chromolith^®^ HighResolution RP-18e column (4.6 × 25 mm) (Merck KGaA, Germany); 750 µL polypropylene autosampler vials with polyethylene snap caps (Thermo Fisher Scientific, Waltham, MA, USA); tip ejector variable volume micropipettes (BOECO, Hamburg, Germany) and pipette tips (Fisher Scientific, Waltham, MA, USA); HyperSep C18 cartridge (Thermo Fisher Scientific).

Working solutions for the HPLC mobile phase gradient were as follows: 20 mM ammonium acetate buffer/acetonitrile (95:5), pH 4 and 100% HPLC acetonitrile. For the solid phase extraction (SPE), working solutions were as follows: Solvent for SPE column regeneration: 100% acetonitrile and 100% MeOH. The acid wash solution was 0.1 M formic acid. 

The internal standard spiking solution was prepared by diluting PFAS (10 ng/mL) in water/methanol (50/50).

#### 2.2.3. Equipment and Instrumentation

A vanquish extractor was equipped with an autosampler and HPLC pump run using the Chromeleon software program (Thermo Fisher). Additionally, we used the following equipment and instrumentation: Orbitrap Exploreris/TSQ Quantis plus triple quadrupole mass spectrometer (Thermo Fisher); CV 2000 liquid handling system (Thermo Scientific); Sartorius Genius Series ME models electronic analytical and semi-microbalances (Sartorius AG, Goettingen, Germany); Sartorius top-loading balance (Sartorius AG, Goettingen, Germany); pH meter (AB 15 pH Meter, Fisher Scientific); vortex mixer (VM 300, Germany Industrial Corp, Taipei, Taiwan).

#### 2.2.4. Automated SPE 

The SPE run of each sample began with the conditioning of a HyperSep C8-SE (7 µM) (or HyperSep C18) cartridge with HPLC-grade acetonitrile (2 mL) and 0.1 M formic acid (2 mL). Afterward, 500 µL of the sample (containing 50 µL serum) injected into the 1 mL sample loop was loaded onto the SPE column using 2 mL 0.1 M formic acid with 1 mL/min flow rate. Next, the SPE column was washed with 2 mL 90% 0.1 M formic acid/10% acetonitrile. The time of the SPE cleanup (including injection time) was 10 min long. 

#### 2.2.5. HPLC Configuration 

The HPLC pump was operated at a 1000 µL/min flow rate with 95% of 20 mM ammonium acetate (pH 4) and 5% of acetonitrile as a mobile phase A and 100% acetonitrile as mobile phase B. The analytes were separated from each other and other extracted components on two Chromolith^®^ HighResolution RP-18e columns (4.6 × 100 mm) preceded by a Chromolith^®^ HighResolution RP-18e (5 × 4.6 mm) guard column and a Chromolith^®^ HighResolution RP-18e (4.6 × 25 mm) column or equivalent. To delay the elution of the PFAS contaminants leaching out from Teflon parts of the HPLC pump, a 4.6 mm × 25 mm Chromolith^®^ HighResolution RP-18e column was inserted between the HPLC pump and the right clamp valve. 

#### 2.2.6. Mass Spectrometer Configuration

Detection of the target analytes was conducted on the TSQ Quantis plus triple quadrupole mass spectrometer or equivalent in the negative ion electrospray ionization (ESI) mode. The TIS ionization source is a variant of the electrospray source and was used here to convert liquid phase ions into gas phase ions. 

Alternatively, automatic sample preparation can be completed following this approach using the CV 2000 liquid handling system.

#### 2.2.7. Instrumental Analysis

We used an online SPE high-performance liquid chromatography tandem MS (SPE-HPLC-MS/MS) method. Briefly, 100 μL of serum (accordingly 10 μL of milk) were mixed with 0.1 M formic acid. Then, internal standards were added (C2-perfluorooctanoic acid (PFOA) and C4-perflucorooctane sulfonic acid (PFOS)) and injected by the SPE-HPLC system to a C18 cartridge. After washing, the target analytes were eluted to a C8 HPLC column (BETASIL C8 column, Thermo Fisher Scientific) for separation. The eluate was then introduced to the MS/MS (API 4000 QTrap, ABSciex) for multiple-reaction-monitoring (MRM) analysis. 

#### 2.2.8. Quality Control and Assurance

The calibration curve was linear for all analytes (analytes were quantified using a calibration curve constructed for each batch: regression coefficients of 0.98 to 0.99 were generally obtained. The limit on the linearity was determined by the highest standard analyzed in the method. Concentrations below the method LOD (or the concentration of the lowest standard in the calibration curve) were reported as not detected.

The limits of detection (LOD) and range—LOD (range)—for each analyte in serum were 0.1 ng/mL (0.1–80 ng/mL) for PFOA; 0.1 ng/mL (0.1–140 ng/mL) for PFOS; and 0.05 ng/mL (0.05–30 ng/mL) for PFHpA, PFHxS, PFDA and PFNA. The LOD in milk was 0.6 ng/L for all PFAS. The LOQ was calculated as 0.3 ng/mL.

The accuracy of the method was determined by enriching serum samples with known concentrations of PFAS and comparing the calculated and expected concentrations. The measurements were taken at 3 different concentrations ranging between 3×LOD and 30 ng/L, and the recovery was 80–120% 

The total relative standard deviations were ≤12% for the precision calculation. 

### 2.3. Statistical Analysis

Continuous variables were determined as means and standard deviations while categorical variables were represented as frequencies and percentages. Regarding PFAS levels in maternal serum, given that more than 60% of the values were not detected, a multiple imputation (MI) analysis was executed, where the upper bound was fixed as the limit of detection (LOD) [33]. Food groups were divided in two categories based on the median consumption portions per week. Geometric means (GM) (log10 of quantitative variable) were used upon need because of the non-normality of distribution for some variables. Since PFAS congeners showed biphasic non-normal distribution, and given that we tried many transformations that did not give a satisfactory normal distribution, we dichotomized them by dividing them into two groups: high and low levels [34]. Regarding PFAS congeners, given that the data followed a distribution pattern that was either lower than the LOD (not detected) or higher than the LOD (detected), the 2 groups were chosen accordingly, as higher and lower than the LOD of each pollutant. Concerning ΣPFASs that is the total sum of all congeners, given that it also followed a dichotomized pattern, the chosen cut-off was the median. Multivariate logistic regression was performed with categorical PFAS levels as dependent variable and sociodemographic, anthropometric, environmental and dietary variables as independent variables. The backward method was adopted and the models with the highest R square values were selected. The backward method is a sequential procedure used in multiple regression. “First, all of the predictors are put into the model. Then, the procedure determines which of the predictors makes the least contribution. That predictor is hence deleted” [34]. Independent variables that yielded a significant *p*-value in the logistic regression were also tested for interaction with fish/shellfish consumption. To assess the effect on newborn anthropometry, multivariate linear regression was performed with dependent variables being Z scores of newborns anthropometric measurements, and PFAS levels as predictors in addition to adjusting for other confounding variables. Due to the substantially high proportion of not detected PFAS levels in cord serum, we could not proceed with the analysis. Regarding human milk, given the low sample size, only descriptive and cross-tabs analysis was performed. Confidence interval of 95% was used and *p*-value ≤ 0.05 was considered significant. Statistical tests were performed using IBM SPSS Statistics for Windows, Version 20, IBM Corp., Armonk, NY, USA. 

## 3. Results

### 3.1. PFAS Levels in Maternal Serum, Cord and Milk

#### 3.1.1. Maternal PFAS Serum Levels

PFAS concentrations are presented in Table 1. The percentage of detection for PFHpA, PFOA, PFHxS and PFOS ranged from 36.3 to 37.7%. In all maternal serum samples, PFNA and PFDA levels were below the LOD. The highest contribution to ΣPFASs was for PFOA and PFOS.

#### 3.1.2. PFAS Cord Serum Levels

PFAS were not detected in cord serum in the vast majority of the samples. The percentage of detection was 13.6% for PFHpA, PFOA, PFHxS and PFOS (8 samples out of 59) (Table 2)

#### 3.1.3. PFAS Human Milk Levels 

Table 3 represents PFAS concentrations in human milk. The percentage of detection was 56.1%. The highest contributor to ΣPFASs was PFOS. 

#### 3.1.4. Correlation between Human Milk and Maternal Serum PFAS Levels

Table S1 shows the correlation between PFAS concentrations in human milk and maternal serum. It ranged between 0.336 and 0.382 (*p* < 0.05). The ratio of milk to serum levels of PFAS were as follows: (median/95th percentile) 0.005/1.373 for ΣPFASs, 0.002/0.193 for PFHpA, 0.002/0.866 PFOA, 0.023/1.824 for PFHxS, and 0.004/2.063 for PFOS.

### 3.2. Association between PFAS Levels and Determinants 

Table S2 in supplementary material represents the bivariate associations across two categories of maternal total PFAS serum levels (low vs. high) with sociodemographic, anthropometric, environmental and dietary maternal characteristics.

Multivariate logistic regression for maternal ΣPFASs serum levels is presented in Table 4 and for the separate PFAS compounds in Tables S3–S6. Fish and shellfish consumption, vicinity to illegal incineration, and having a university level education versus high school or less were all associated with an almost twice higher risk of elevated PFHpA, PFOA, PFHxS and PFOS serum levels. These results were obtained after adjustment for age, parity, pre-pregnancy BMI, GWG, crowding index, having been breastfed, geographical vicinity to landfills, geographical vicinity to factories, smoking, passive smoking, alcohol consumption, red meat consumption, poultry consumption, dairy products consumption, eggs consumption and fruits consumption. The Hosmer–Lemeshow test was also run for sample adequacy to data, and it was not significant.

Table 5 shows the bivariate associations between dietary intake and ΣPFASs human milk levels. Results for separate PFASs compounds are available in Table S7 in the supplementary material. Significantly higher PFAS levels were observed along with a higher consumption of dairy products and eggs. Moreover, 5 out of 35 women usually drank tap water, and they all had high PFAS levels. 

### 3.3. Effect of PFAS Maternal Serum Levels on Newborn Anthropometry 

Multivariate linear regression showed a significant negative association between PFHpA exposure categories and Z-scores of “weight-for-length”, with adjustment for confounding variables (Table 6). ΣPFASs and the other PFAS compounds showed a similar trend but were only near significance (*p*-value ~ 0.057–0.071) (Tables S8–S11 in the Supplementary Material). Other newborn anthropometric measurements were similarly analyzed, but did not yield any significant associations with PFAS levels. These anthropometric measurements include weight-for-age, length-for-age and head circumference-for-age (Tables S12–S16 in Supplementary Material).

## 4. Discussion

The current study established an estimate of the serum levels of six PFAS compounds among Lebanese pregnant women, their distribution in cord serum and human milk, as well as their determinants and their impact on newborn anthropometry. This provides an idea of the magnitude of exposure of fetuses to these pollutants, given that these compounds could reach them through transplacental transfer [21], despite the fact that PFAS were not detected in the majority in cord serum. These findings display a need for urgent attention and interventions to reduce the environmental exposure among subgroups of the population with higher PFAS levels. 

### 4.1. Levels of PFAS and Comparison to Guidance Values 

When comparing the PFAS maternal serum levels (95th percentile) to the newly released “international human biomonitoring (i-HBM) working group’s health-based guidance value (HB2GV)” [35,36,37], we observed that PFOA and PFOS levels were much higher than the HBM-I and HBM-II values but lower than the reference dose values (RfD). HBM-II values are 5 ng/mL for PFOA and 10 ng/mL for PFOS among women at childbearing age, and 10 ng/mL for PFOA and 20 ng/mL for PFOS among all other population groups [37]. As per the PFHpA and PFHxS, they showed levels much lower than the guidance values (GVs). PFNA and PFDA levels were also much lower since they were not detected in maternal serum. The results obtained with PFOA and PFOS are of major importance since a value above the HBM-II indicates that there is an increased risk for adverse health effects and, therefore, there is an urgent need to diminish exposure for a substantial proportion of the society [36]. As a matter of fact, the high levels obtained with PFOA and PFOS in the present study surpassed those obtained at the fourth—and highest—quartile in a study conducted in the USA, where they were associated with a higher prevalence of current thyroid disease [38]. The fact that PFOA and PFOS levels in our study showed a biphasic distribution is of major interest, as women either had low levels of exposure to PFAS, or a relatively high level (higher than the HBM-II levels), without intermediate values. The reasons for this distribution are worth being investigated more closely, and can be due to specific sources of contamination, such as fish and shellfish consumption. This might reflect a lifestyle usually adopted by only a part of the population (more details are discussed below). 

In comparison with studies solely conducted among pregnant women, PFOA, PFOS and PFHpA serum levels—at the 95th percentile—were higher than those detected in the USA [12,18] and in Shanghai [14]. Serum levels of PFOS and PFHpA were additionally higher than those reported in Sweden [16]. PFOS and PFOA human milk concentrations scored similar to higher concentration levels as compared to other studies conducted in Spain [39] and the United States [40]. Compared to the study conducted in Lebanon [25], our study showed relatively lower concentrations of PFOA but higher concentrations of PFOS. The comparison of PFAS levels to international studies should be done carefully, given that the percentage of detection (in serum) ranged from 36.3 to 37.7%. Moreover, the range of values for PFAS levels in our study was narrower, and levels were gathered around two main categories of distribution (high vs. low). The aforementioned reasons generated less variability across population categories, thus affecting correlations observed in this study. Data about serum PFAS levels in the region is scarce, especially among pregnant women. Nevertheless, a study was conducted in Saudi Arabia and PFAS levels were measured among 208 adults, with a percentage of detection >80% [41]. In our study, this percentage is clearly much lower, despite the maximum levels of PFAS being higher. Low detection levels could be explained by the fact that our study sample was not evenly exposed to contaminated sources of PFAS, as previously mentioned. More details on the factors that predicted higher levels of PFAS are discussed below. Moreover, many studies measured PFAS among highly exposed populations, in highly contaminated areas (i.e., Italy [42]), which was not the case in the current study.

### 4.2. Partitioning of PFAS across Matrices 

Our study showed a disproportionate distribution of PFAS across biological matrices with highest PFAS levels in maternal serum, lower—but detected—levels in human milk, and almost not detected levels in cord serum. Scientific evidence reported that PFAS levels are 1–3 times lower in cord blood than maternal serum, and 10 times lower in human milk than maternal serum [43]. Generally speaking, PFAS levels in biological matrices are highly influenced by physiological dynamics, their physicochemical properties, and the transport proteins they are usually bound to [44]. While the most important PFAS-related transport protein among humans is albumin [45], there are several other molecules such as transmembrane transport proteins [44]. PFAS chain length plays a major role in determining the binding affinity to transport protein, therefore affecting PFAS levels in biological matrices. In general, the longer the PFAS carbon chain, the higher the binding affinity [44]; this—in part—could explain why PFDA was not detected in any of the matrices in contrast to the other PFAS molecules. The PFAS ratio of milk to maternal serum coincides with the findings that PFAS levels in human milk are generally lower than those in maternal serum [46]. An interesting observation is that while PFNA was not detected in maternal serum, it was detected in human milk. Moreover, while PFHxS ranked third in level in maternal serum among all PFAS after PFOS and PFOA, it ranked second after PFOS in human milk. In fact, two mechanisms of transfer of PFAS from blood to human milk are possible. The first one is similar to the fatty acids through the mammary epithelial membrane, given their similarities in structure. The second one is through the same membrane but involves diffusion for smaller molecules and pinocytosis for the larger ones. The second process could explain the presence of shorter chain PFAS molecules in human milk as compared to larger ones [46]. Regarding cord serum, the fact that PFAS levels were not detected in majority shows the extent to which PFAS levels are influenced by physiological factors, including transport proteins. This does not mean that PFAS are not crossing the placental barrier at all [47]. Recent findings added to the toxicity-inducing mechanisms of endocrine-disrupting chemicals (EDCs) among pregnant women, by proving that EDCs trigger the activation of the P2X7 receptor and mitochondrial alteration in human placental cells, therefore potentially affecting the health of the mother and the fetus [48]. Therefore, the results of our study could signal the presence of a threshold for PFAS in serum, above which transfer to cord serum and other physiological mechanisms are triggered. Recent experimental studies have estimated a “point of departure” of EDCs mixtures, which is a toxicological measure of no-effect concentration [49]. The aforementioned analysis could be seen positively with regards to protecting the fetus from exposure to high levels of PFAS. Moreover, this highlights the role the placenta could be playing in protecting the fetus from one side and in being an endocrine gland regulating both fetal growth and maternal health during pregnancy from the other side [50]. 

### 4.3. Determinants of PFAS Levels

Associations with multiple factors showed that fish and shellfish consumption, vicinity to illegal incineration, and higher education were major determinants of exposure to PFAS in the present sample.

One of the primary routes of exposure to PFAS is dietary [4], with the consumption of fish and shellfish being the major source [7]. In fact, PFAS reach river and sea water through sources of contamination and bioaccumulate in fish [10]. Our findings showed a remarkable association between fish and shellfish consumption and PFAS serum levels, which is in line with other studies among pregnant women [6,51], women of childbearing age [52] and adults [53]. In contrast, a study by Kingsley et al. did not exhibit such an association, and it was suggested to be related to the low fish consumption among the pregnant women of the study [54]. In Lebanon, fish is mostly imported—live, fresh, and frozen—from countries such as Turkey, Vietnam and Egypt [55]. In fact, PFAS was detected in these countries, specifically in air dust and food packaging in Egypt [56], in tap water in Turkey [57] and in freshwater fish in Vietnam [58], although levels were below the values considered safe/tolerable in all these studies. Moreover, another source—in Lebanon—that contributes to a lesser extent to fish production (mostly trout) is aquaculture, through family owned farms [55]. In general, fish points of sale include ports auctions, farms, some restaurants, and supermarkets [55,59]. To date, no studies have been conducted in Lebanon to measure PFAS in fish. Nevertheless, an important aspect to consider here is that the level of PFAS present in fish itself is influenced by several biotic (seasonal changes, diet, reproduction, health status) and abiotic factors (salinity, temperature) leading to physiological changes, among which are PFAS-related proteins, thus eventually affecting the actual level of PFAS in animal tissue [44]; this should be taken into account when interpreting results. In addition, another factor that might be affecting PFAS exposure from fish and shellfish and that is worth being investigated is the effect of packaging used for fish and shellfish, namely during transportation for import, as PFAS has been shown to migrate from food contact material to food [60,61].

Other than fish and shellfish, we observed higher human milk concentrations of PFOA, PFOS, PFHpA, PFNA and PFHxS with a higher consumption of eggs and dairy products among lactating women. While these results should be carefully considered, it should be noted that PFAS transfer to eggs among animals has been studied before, and it is considered as a maternal off-loading of PFAS [44]. In particular, a study conducted in Belgium nearby a fluorochemical plant showed that home-grown eggs were contaminated with PFAS, and constitute to humans an ‘important exposure pathway’ [62]. The study by Hassan et al. did not exhibit a similar association, but it showed that higher PFOA milk levels were observed with higher consumption of “bread and pasta”, “meat and chicken” and white tubers, and higher PFOS levels were observed with higher consumption of cereals [25]. Regarding the consumption of other food groups, although red meat, chicken, and fruits were shown to be food sources of PFAS [5,6,7,8,27], this was not exhibited in the current study. It is worth noting that the European Commission recently adopted amendments of the regulation number 1881/2006 as regards maximum levels of PFAS in certain foodstuffs, notably eggs, fish, shellfish, meat, poultry and offal [63]. 

Another major source of contamination by PFAS is contaminated drinking water. In a study conducted in “Ronneby-Sweden”, in an area that was subject to AFFF-contamination of drinking water, levels of all PFAS compounds were generally higher than levels in the current study [64]. Among the lactating women of our study sample, only 5 were drinking tap water and all had higher human milk PFAS levels. Given that the sample is small and that this information was not collected among the wider sample, further studies should be conducted in Lebanon to examine PFAS in drinking water and groundwater.

In addition to dietary exposure, an environmental factor—i.e., vicinity to illegal incineration—also showed an association with PFAS serum levels in the present study. In fact, open-burning of waste has been previously linked with PFAS release. Mothers residing in “Guiyu–China”, where e-waste recycling through open-burning is common, had higher PFOA serum levels than their counterparts in the control group [65]. This is distinct from the incinerators used for the disposal of waste containing PFAS; while this process has been effective to some extent in reducing long-chain PFAS, studies have shown that it could release other harmful compounds (such as fluorinated greenhouse gases and products of incomplete combustion), and that “some PFAS may remain in the incinerator ash” [66]. In Lebanon, municipal solid waste is mostly landfilled (51%) and open-dumped (32%), with only 17% recycled or composted [67]. Moreover, a waste crisis occurred in 2015 in response to the sudden closure of a major landfill, and the subsequent suspension of waste collection, which resulted in the anarchic open burning of domestic waste [68]. This is of particular importance because Lebanon’s geographical position on the Eastern basin of the Mediterranean Sea, as well as the flow of two of its rivers to neighboring countries, increases the risk of exchange in contamination to this surrounding.

Regardless of incineration, studies have been documenting to date the occurrence of PFAS in landfills and subsequently in landfills leachates [69], despite removal and disposal attempts of PFAS-containing waste [66]. Moreover, one major source of PFAS contamination is industrial wastewater as well as municipal and industrial wastewater treatment plants [66,70]. Although in the present study, we did not observe an association with vicinity to landfills or factories, the effect of landfills leachates and industrial wastewater could still reach and pollute underground water and soil, hence food crops and livestock [71].

Although there is no clear explanation to the association obtained with education, these findings are consistent with other studies that similarly reported this association [72,73,74], including a recent study by Eick et al. where “levels of PFOA, PFOS, PFNA and PFHxS were higher among women with a college or graduate education compared to those with less than a college degree” [72]. Nevertheless, this association was in contrast with a study performed by Sagiv et al. among 1645 pregnant women [75]. A higher education could be linked to a higher socioeconomic status, more outgoing and other lifestyle differences, and maybe a different geographical location (living in urban areas more than rural…). 

No associations were obtained between age, parity, pre-pregnancy BMI, smoking, alcohol consumption and PFAS serum levels, although they were observed in other studies [15,17,54,75]. This could be explained by the low variability between the sample categories, due to the narrow range of distribution of PFAS as compared to other studies. 

### 4.4. Impact of PFAS on Newborn Anthropometry

Our study showed an association between higher PFHpA maternal serum levels and a lower weight-for-length Z-score, while no associations were found with other congeners. No statistically significant associations were obtained with other anthropometric newborn outcomes. As a matter of fact, findings in the literature are inconsistent. Previous studies did not find strong correlations between PFAS levels and birth weight [11,18,76]. Conversely, negative associations were reported elsewhere with birth weight [77,78], length, and head circumference [77]. Many findings have revealed that prenatal exposure to EDCs mixtures affects birthweight Z-scores, with PFAS being in particular among the main chemicals of concern [79]. In fact, several mechanisms were exhibited to explain how PFAS was associated with lower birth weight, namely through oxidative stress and subsequent expression of PPAR-γ and the following signaling pathways, leading to a reduced adipose tissue weight, caused itself by the hyper-differentiation of pre-adipocytes [80]. Another mechanism is through endocrine and estrogenic effects of PFAS [80]. The fact that a significant association was only obtained with PFHpA—with a trend for other PFAS—could reflect the impact of PFAS molecular weight and carbon chain length, but could also highlight a serum level threshold effect or a protective mechanism from the placenta as previously mentioned. Therefore, this association was not observed with ΣPFASs, given that PFHpA constitutes a low contribution to the total sum of PFAS, especially when compared to PFOA and PFOS. 

### 4.5. Strength and Limitations

This study provided valuable addition to the literature in terms of the exposure of the mother and her child to PFAS. It showed that although certain measures are undertaken internationally to reduce the use of PFAS, substantial actions are still needed to reduce its exposure, namely among critical populations. This is the first study in Lebanon to measure six PFAS compounds in maternal serum, assess their determinants, their impact on newborn anthropometry, as well as their correlation with PFAS measured in cord serum and human milk among a subsample from the same population.

Nevertheless, there are several limitations. The study design is cross-sectional, and there might be residual confounding factors that were not taken into consideration; therefore, other experimental studies are needed to further evaluate the study findings. Moreover, although the present sample involves a critical population, it is not representative of the Lebanese adult population. In addition, sampling happened at childbirth, and did not consider certain physiological and behavioral changes that may occur during pregnancy trimesters. Finally, reporting biases could have impacted information related to dietary intake and vicinity to sources of contamination. 

## 5. Conclusions

The current study allowed us to determine levels of six PFAS compounds among Lebanese pregnant women and to show that serum levels are higher than HBM-I and HBM-II values for PFOA and PFOS. PFHpA and PFHxS levels were remarkable but lower than GVs, while PFDA and PFNA were not detected among our sample. While the six PFAS compounds were not detected in cord serum, five compounds were detected in human milk. Our findings showed that higher PFAS serum levels were observed among pregnant women with higher fish and shellfish consumption, higher educational level and higher vicinity to illegal incineration. Higher PFAS human milk levels were observed with a higher consumption of eggs and dairy products. The positive association with drinking tap water was preliminary but not enough to derive conclusions. In addition, PFHpA serum levels were negatively associated with weight-for-length Z-score for newborns at birth. With research on PFAS continuously showing important breakthroughs on an international level, this study establishes an important basis for future and larger studies in Lebanon, and highlights the importance of examining the interconnection between countries from an environmental perspective. In particular, urgent action is needed to reduce environmental exposure in Lebanon among subgroups of the population with higher PFAS levels.

## Figures and Tables

**Table 1:** PFAS concentrations in maternal serum samples (*n* = 419).

	*n*	%	Mean (±SD)	Min	50th *p*	95th *p*	Max	%Contribution to ΣPFASs Median Levels: Total Sample (Low, High) ^b^
ΣPFASs								
<0.3 ng/mL	267	63.7	0.26 ± 0.01	0.21	0.26	0.28	0.29	-
≥0.3 ng/mL	152	36.3	99.98 ± 8.71	74.42	100.96	111.56	113.34	
Total sample ^a^	419		-	0.21	0.27	109.19	113.34	
PFHpA								15.61 (16.49; 0.31)
<0.05 ng/mL	266	63.5	0.042 ± 0.003	0.031	0.042	0.048	0.049	
≥0.05 ng/mL	153	36.5	0.307 ± 0.035	0.050	0.310	0.340	0.360	
Total sample ^a^	419		-	0.031	0.045	0.340	0.360	
PFOA								35.01 (33.17; 44.46)
<0.10 ng/mL	264	63	0.085 ± 0.008	0.065	0.085	0.097	0.099	
≥0.10 ng/mL	155	37	43.524 ± 7.426	0.100	45.000	50.110	51.250	
Total sample ^a^	419		-	0.065	0.092	49.100	51.250	
PFHxS								16.09 (17.02; 2.08)
<0.05 ng/mL	261	62.3	0.044 ± 0.003	0.035	0.043	0.049	0.049	
≥0.05 ng/mL	158	37.7	2.014 ± 0.431	0.050	2.170	2.301	2.380	
Total sample ^a^	419		-	0.035	0.047	2.270	2.380	
PFOS								35.20 (33.54; 53.17)
<0.10 ng/mL	267	63.7	0.086 ± 0.007	0.065	0.086	0.097	0.100	
≥0.10 ng/mL	152	36.3	53.204 ± 4.516	39.250	54.125	59.735	61.250	
Total sample ^a^	419		-	0.065	0.092	57.800	61.250	

^a^: log was used; ^b^: percentage of contribution to ΣPFASs in the total sample and in the 2 categories of ΣPFASs levels (low and high ΣPFASs); SD = Standard Deviation.

**Table 2:** Cord serum PFAS levels (ng/mL) among a subsample of women (*n* = 59).

	*n* = 59		*n* = 8		
	<LOD *n* (%)	>LOD *n* (%)	Mean (±SD) ^a^	50th *p* ^a^	Max ^a^
PFHpA	51 (86.4)	8 (13.6)	0.30 ± 0.04	0.31	0.35
PFOA	51 (86.4)	8 (13.6)	43.28 ± 5.88	44.85	49.50
PFNA	59 (100)	0.0	-	-	-
PFDA	59 (100)	0.0	-	-	-
PFHxS	51 (86.4)	8 (13.6)	2.00 ± 0.17	1.98	2.20
PFOS	51 (86.4)	8 (13.6)	53.03 ± 6.02	54.60	58.30

LOD = 0.1 ng/mL for PFOA and PFOS; 0.05 ng/mL for PFHpA, PFHxS, PFDA and PFNA. ^a^ Values calculated for 8 samples where PFAS was detected in cord.

**Table 3:** PFAS concentrations in human milk (*n* = 41).

	*n*	%	Mean (±SD)	Min	50th *p*	95th *p*	Max	%Contribution to ΣPFASs Median Levels: Total Sample (Low, High) ^a^
ΣPFAS								-
Low	18	43.9	2.26 ± 0.21	1.88	2.24	-	2.56	
High	23	56.1	284 ± 64.87	174.0	302.5	371.26	373.50	
Total sample	41	100	160.45 ± 149.62	1.88	202.80	361.52	373.50	
PFHpA								5.03 (20.72; 4.65)
<0.06 ng/L	18	43.9	0.44 ± 0.10	0.27	0.45	-	0.58	
≥0.06 ng/L	23	56.1	13.03 ± 3.85	6.80	14.60	18.08	18.10	
Total sample	41	100	7.50 ± 6.94	0.27	7.20	17.97	18.10	
PFOA								20.80 (19.10; 21.66)
<0.06 ng/L	18	43.9	0.43 ± 0.08	0.29	0.42	-	0.56	
≥0.06 ng/L	23	56.1	60.78 ± 13.98	39.00	65.00	79.40	80.00	
Total sample	41	100	34.29 ± 32.05	0.29	42.00	76.90	80.00	
PFNA								0.69 (20.72; 0.64)
<0.06 ng/L	18	43.9	0.48 ± 0.06	0.33	0.47	-	0.59	
≥0.06 ng/L	23	56.1	1.87 ± 0.38	1.20	2.00	2.40	2.40	
Total sample	41	100	1.26 ± 0.75	0.33	1.30	2.39	2.40	
PFHxS								22.72 (19.64; 23.21)
<0.06 ng/L	18	43.9	0.45 ± 0.06	0.34	0.45	-	0.59	
≥0.06 ng/L	23	56.1	67.57 ± 12.6	42.00	71.00	84.20	85.00	
Total sample	41	100	38.10 ± 34.99	0.34	48.00	80.90	85.00	
PFOS								47.25 (20.92; 49.81)
<0.06 ng/L	18	43.9	0.47 ± 0.09	0.32	0.47	-	0.60	
≥0.06 ng/L	23	56.1	141.00 ± 35.04	141.00	147.00	187.40	188.00	
Total sample	41	100	79.30 ± 75.24	0.32	91.00	184.70	188.00	

^a^: percentage of contribution to ΣPFASs in the total sample and in the 2 categories of ΣPFASs levels (low and high ΣPFASs); SD = Standard Deviation.

**Table 4:** Logistic regression for maternal serum ΣPFASs using backward method (*n* = 269).

		95% CI		
	OR ^b^	LB	UB	*p*-Value
ΣPFASs High vs. Low ^a^				
Fish/shellfish consumption ≥ 0.63 vs. <0.63 portion/week ^c,d^	1.90	1.05	3.46	0.034
Vicinity to illegal incinerations: Yes vs. No ^d^	2.03	1.08	3.81	0.029
Education: University level vs. high school or less ^c^	2.07	1.10	3.89	0.025
Age ≥ 30 vs. <30	0.85	0.46	1.58	0.613
Multiparous vs. Primiparous	0.70	0.36	1.36	0.293
Pre-pregnancy BMI ≥ 25 vs. <25 kg/m^2^	0.98	0.51	1.86	0.944
GWG				
Normal vs. Inadequate	1.19	0.59	2.42	0.630
Excessive vs. Inadequate	1.01	0.48	2.14	0.983
Crowding index: ≥1 vs. <1	0.96	0.45	2.09	0.927
Breastfed: Yes vs. No	1.02	0.38	2.70	0.971
Vicinity to factories: Yes vs. No	0.71	0.39	1.28	0.256
Vicinity to landfills: Yes vs. No	1.00	0.53	1.90	0.994
Ever smoking: Yes vs. No	0.72	0.40	1.32	0.292
Passive smoking: Yes vs. No	1.51	0.74	3.06	0.255
Alcohol: Yes vs. No	0.44	0.19	1.06	0.067
Red meat consumption: ≥1.94 vs. <1.94 portions/week	1.60	0.91	2.83	0.105
Dairy products consumption: ≥11.5 vs. <11.5 portions/week	0.72	0.40	1.29	0.274
Poultry consumption: ≥2 vs. <2 portions/week	1.26	0.71	2.24	0.422
Eggs consumption: ≥1.4 vs. <1.4 portions/week	0.99	0.55	1.78	0.977
Fruits consumption: ≥10.5 vs. <10.5 portions/week	1.10	0.61	1.97	0.746

BMI: body mass index; GWG: gestational weight gain; CI: confidence interval; OR: Odds Ratio; LB: lower bound; UB: upper bound. Test: Logistic regression; *p* ≤ 0.05 was considered significant. ^a^ Threshold of ΣPFAS was determined according to distribution. ^b^ Variables entered as predictors were age, parity, pre-pregnancy BMI, GWG, crowding index, education, having been breastfed, geographical vicinity to landfills, geographical vicinity to factories, illegal incinerations, smoking, passive smoking, alcohol consumption, fish/shellfish consumption, red meat consumption, poultry consumption, dairy products consumption, eggs consumption, and fruits consumption. ^c^ p-interaction (fish/shellfish consumption × education) = 0.984. ^d^ p-interaction (fish/shellfish consumption × vicinity to illegal incineration) = 0.569.

**Table 5:** Association between dietary intake, use of tap water and ΣPFAS human milk levels (*n* = 35).

		PFAS Categories				
		Low (*n* = 18) *n*; %		High (*n* = 21) *n*; %		*p*-Value ^a^
ΣPFAS						
Dairy Products	<1.5 portions/day	14	77.8%	8	38.1%	0.013
	≥1.5 portions/day	4	22.2%	13	61.9%	
Eggs ^b^	<1.68 portion/week (Median)	13	72.2%	6	28.6%	0.007
	>1.68 portion/week (Median)	5	27.8%	15	71.4%	
Use of Tap Water	Yes	0	0.0%	5	27.8%	0.019
	No	17	100.0%	13	72.2%	

^a^ statistical test: Chi-square. ^b^ significant in non-parametric test, *p* for all PFAS is 0.013. *p* ≤ 0.05 was considered significant.

**Table 6:** Multivariate linear regression for Z-scores of newborns weight-for-length at birth with maternal serum PFHpA as predictor (*n* = 243).

		95% CI		
	Unst. Β ^b^	LB	UB	*p*-Value
Weight-for-length				
PFHpA: High vs. Low ^a^	−0.035	−0.070	0.000	0.047
Age: ≥30 vs. <30 years	−0.008	−0.043	0.027	0.664
Multiparous vs. Primiparous	0.015	−0.024	0.054	0.450
Pre-pregnancy BMI: ≥25 vs. <25 kg/m^2^	−0.030	−0.068	0.008	0.123
GWG	0.011	−0.011	0.032	0.334
Pregnancy weight loss from restrictive diet: Yes vs. No	0.028	−0.051	0.106	0.490
Pre−pregnancy weight loss from restrictive diet: Yes vs. No	−0.007	−0.051	0.036	0.739
Passive smoking: Yes vs. No	0.001	−0.040	0.042	0.967
Smoking: Yes vs. No	0.006	−0.029	0.041	0.745
Gestational age (in weeks)	0.010	−0.003	0.023	0.122
Crowding index: >1 vs. ≤1	−0.035	−0.080	0.010	0.128
Education University vs. high school or less	0.039	0.002	0.076	0.039

BMI: body mass index; Unst. β: unstandardized beta; CI: confidence interval; LB: lower bound; UB: upper bound; GWG: gestational weight gain. Test: linear regression; *p* ≤ 0.05 was considered significant. ^a^ Threshold was determined according to distribution. ^b^ Variables included as predictors were age, parity, pre-pregnancy BMI, GWG, pre-pregnancy and pregnancy weight loss from restrictive diet, crowding index, education, smoking, and passive smoking.

## Data Availability

The data presented in this study are available on request from the corresponding author. The data are not publicly available due to privacy reasons.

## Supplementary Materials

The following supporting information can be downloaded at: https://www.mdpi.com/article/10.3390/toxics11050455/s1, Table S1. PFAS maternal serum–human milk correlation (*n* = 38); Table S2. Characteristics of the study participants with regards to maternal total PFAS serum levels categories (*n* = 269); Table S3. Logistic regression for maternal serum PFHpA using backward method (*n* = 269); Table S4. Logistic regression for maternal serum PFOA using backward method (*n* = 269); Table S5. Logistic regression for maternal serum PFHxS using backward method (*n* = 269); Table S6. Logistic regression for maternal serum PFOS using backward method (*n* = 269); Table S7. Association between dietary intake, use of tap water, and PFAS human milk levels (n = 35); Table S8. Multivariate Linear Regression for Z-scores of newborns weight-for-length at birth with maternal serum ΣPFAS as predictor (*n* = 243); Table S9. Multivariate Linear Regression for Z-scores of newborns weight-for-length at birth with maternal serum PFOA as predictor (*n* = 243); Table S10. Multivariate Linear Regression for Z-scores of newborns weight-for-length at birth with maternal serum PFHxS as predictor (*n* = 243); Table S11. Multivariate Linear Regression for Z-scores of newborns weight-for-length at birth with maternal serum PFOS as predictor (*n* = 243); Table S12. Multivariate Linear Regression for Z scores of newborns anthropometric measurements at birth with PFHpA as predictor (*n* = 243); Table S13. Multivariate Linear Regression for Z scores of newborns anthropometric measurements at birth with PFOA as predictor (*n* = 243); Table S14. Multivariate Linear Regression for Z scores of newborns anthropometric measurements at birth with PFHxS as predictor (*n* = 243); Table S15. Multivariate Linear Regression for Z scores of newborns anthropometric measurements at birth with PFOS as predictor (*n* = 243); Table S16. Multivariate Linear Regression for Z scores of newborns anthropometric measurements at birth with ∑PFAS as predictor (*n* = 243).
