# Supplementary material for: Maternal Serum, Cord and Human Milk Levels of Per- and Polyfluoroalkyl Substances (PFAS), Association with Predictors and Effect on Newborn Anthropometry
Source: Toxics. 2023 May 14;11(5):455. doi: 10.3390/toxics11050455 (PMC10223184; doi:10.3390/toxics11050455)
Supplement: Supplementary file 1 [file toxics-11-00455-s001.zip › toxics-2329776-supplementary.pdf]

Maternal serum, cord and human milk levels of Per- and Polyfluoroalkyls (PFAS), association with predictors and effect on newborn anthropometry

Supplementary Material

Table of content

Content	Page
**Table S1.** PFAS maternal serum – human milk correlation (n=38)	S2
**Table S2.** Characteristics of the study participants with regards to maternal total PFAS serum levels categories (*n* = 269)	S2-3
**Table S3**. Logistic regression for maternal serum PFHpA using backward method (N=269)	S4
**Table S4**. Logistic regression for maternal serum PFOA using backward method (N=269)	S4-5
**Table S5**. Logistic regression for maternal serum PFHxS using backward method (N=269)	S5-6
**Table S6**. Logistic regression for maternal serum PFOS using backward method (N=269)	S6-7
**Table S7.** Association between dietary intake, use of tap water, and PFAS human milk levels (n=35)	S8-9
**Table S8**. Multivariate Linear Regression for Z-scores of newborns weight-for-length at birth with maternal serum ∑PFAS as predictor (n=243)	S10
**Table S9**. Multivariate Linear Regression for Z-scores of newborns weight-for-length at birth with maternal serum PFOA as predictor (n=243)	S10-11
**Table S10**. Multivariate Linear Regression for Z-scores of newborns weight-for-length at birth with maternal serum PFHxS as predictor (n=243)	S11
**Table S11**. Multivariate Linear Regression for Z-scores of newborns weight-for-length at birth with maternal serum PFOS as predictor (n=243)	S12
**Table S12.** Multivariate Linear Regression for Z scores of newborns anthropometric measurements at birth with PFHpA as predictor (n=243)	S13-14
**Table S13.** Multivariate Linear Regression for Z scores of newborns anthropometric measurements at birth with PFOA as predictor (n=243)	S15-16
**Table S14.** Multivariate Linear Regression for Z scores of newborns anthropometric measurements at birth with PFHxS as predictor (n=243)	S17-18
**Table S15.** Multivariate Linear Regression for Z scores of newborns anthropometric measurements at birth with PFOS as predictor (n=243)	S19-20
**Table S16**. Multivariate Linear Regression for Z scores of newborns anthropometric measurements at birth with ∑PFAS as predictor (n=243)	S21-22

Table S1. PFAS maternal serum – human milk correlation (n=38)

	Maternal serum PFAS levels				
	∑PFAS (ng/mL)	PFHpA (ng/mL)	PFOA (ng/mL)	PFHxS (ng/mL)	PFOS (ng/mL)
Human milk PFAS levels					
∑PFAS (ng/L)	0.360*	0.349*	0.364*	0.369*	0.356*
PFHpA (ng/L)	0.349*	0.336*	0.352*	0.357*	0.347*
PFOA (ng/L)	0.348*	0.337*	0.353*	0.357*	0.344*
PFNA (ng/L)	0.369*	0.356*	0.373*	0.377*	0.366*
PFHxS (ng/L)	0.372*	0.361*	0.377*	0.382*	0.368*
PFOS (ng/L)	0.358*	0.347*	0.363*	0.367*	0.354*

Statistical Test: Pearson correlation
* correlation is significant at the 0.05 level (2-tailed)

Table S2. Characteristics of the study participants with regards to maternal total PFAS serum levels categories (*n* = 269)

		∑ PFASs				
		Low		High		
		Count	%	Count	%	*p*-value
**Sociodemographic and other maternal characteristics**						
Region of Recruitment	Beirut / Mount-Lebanon	61	35.9%	37	37.4%	0.000
	Beqaa	109	64.1%	34	34.3%	
	Sidon/Nabatieh	0	0.0%	28	28.3%	
Age	20-30 years	79	46.5%	53	53.5%	0.264
	30-40 years	91	53.5%	46	46.5%	
Parity	Primiparous	56	32.9%	43	43.4%	0.085
	Multiparous	114	67.1%	56	56.6%	
Education	High school or less	78	45.9%	29	29.3%	0.007
	University level	92	54.1%	70	70.7%	
Crowding Index[a]	≤1	132	77.6%	83	83.8%	0.22
	> 1	38	22.4%	16	16.2%	
Breastfed	Yes	152	90.5%	86	90.5%	0.989

	No	16	9.5%	9	9.5%	
**Anthropometric characteristics**						
BMI	< 25 kg/m^2	107	62.9%	64	64.6%	0.779
	≥ 25 kg/m^2	63	37.1%	35	35.4%	
GWG[b]	Inadequate	61	35.9%	29	29.3%	0.519
	Adequate	52	30.6%	35	35.4%	
	Excessive	57	33.5%	35	35.4%	
**Environmental characteristics**						
Geographical vicinity to factories	No	90	54.5%	58	58.6%	0.522
	Yes	75	45.5%	41	41.4%	
Geographical vicinity to landfills	No	85	50.0%	45	45.5%	0.472
	Yes	85	50.0%	54	54.5%	
Illegal incineration	No	87	51.2%	35	35.4%	0.012
	Yes	83	48.8%	64	64.6%	
**Behavioral characteristics**						
Passive smoking	No	39	22.9%	21	21.2%	0.743
	Yes	131	77.1%	78	78.8%	
Ever smoking	No	95	55.9%	59	59.6%	0.553
	Yes	75	44.1%	40	40.4%	
Alcohol consumption	Yes	30	17.6%	13	13.1%	0.330
	No	140	82.4%	86	86.9%	
**Dietary characteristics**						
Fish/Shellfish	<0.63 portions/week	91	53.5%	37	37.4%	0.011
	≥ 0.63 portions/week	79	46.5%	62	62.6%	
Red meat	<1.94 portions/week	93	54.7%	41	41.4%	0.035
	≥ 1.94 portions/week	77	45.3%	58	58.6%	
Poultry	< 2 portions/week	82	48.2%	40	40.4%	0.213
	≥ 2 portions/week	88	51.8%	59	59.6%	
Eggs	<1.4 portions/week	88	52.1%	45	45.9%	0.332
	≥ 1.4 portions/week	81	47.9%	53	54.1%	
Dairy Products	< 11.5 portions/week	78	45.9%	50	50.5%	0.464
	≥ 11.5 portions/week	92	54.1%	49	49.5%	
Fruits	<10.5 portions/week	80	47.1%	44	44.4%	0.678
	≥ 10.5 portions/week	90	52.9%	55	55.6%	

SD: standard deviation; BMI: body mass index; GWG: gestational weight gain;
Statistical test: Chi-square; $p<0.05$ is significant;
[a]Crowding index was defined as the total number of co-residents per household, excluding the newborn infant, divided by the total number of rooms, excluding the kitchen and the bathrooms
[b]GWG was categorized based on the Institute of Medicine (IOM) guidelines (IOM and NRC, 2009)

Table S3. Logistic regression for maternal serum PFHpA using backward method (N=269)

	95% CI			
	**OR[b]**	**LB**	**UB**	**p- value**
**PFHpA High vs Low[a]**				
Fish / Shellfish consumption ≥0.63 vs <0.63 portion/week[c, d]	2.01	1.11	3.66	0.022
Vicinity to illegal incinerations Yes vs No[d]	1.88	1.00	3.53	0.048
Education University level vs High school or less[c]	2.17	1.16	4.09	0.016
Age ≥30 vs <30	0.86	0.46	1.60	0.638
Multiparous vs Primiparous	0.74	0.38	1.42	0.360
Pre-pregnancy BMI ≥25 vs <25 kg/m^2	0.96	0.50	1.83	0.900
GWG				
Normal vs Inadequate	1.13	0.56	2.29	0.739
Excessive vs Inadequate	0.96	0.46	2.04	2.04
Crowding index ≥1 vs <1	0.92	0.42	1.98	1.98
Breastfed Yes vs No	1.04	0.39	2.76	0.938
Vicinity to factories Yes vs No	0.68	0.38	1.23	0.200
Vicinity to landfills Yes vs No	1.10	0.58	2.08	0.778
Ever smoking Yes vs No	0.70	0.39	1.29	0.255
Passive smoking Yes vs No	1.56	0.77	3.18	0.215
Alcohol Yes vs No	0.43	0.18	1.03	0.058
Red meat consumption ≥ 1.94 vs <1.94 portions/week	1.56	0.88	2.76	0.125
Dairy products consumption ≥11.5 vs <11.5 portions/week	0.74	0.41	1.32	0.302
Poultry consumption ≥ 2 vs <2 portions/week	1.22	0.69	2.16	0.491
Eggs consumption ≥1.4 vs <1.4 portions/week	0.93	0.52	1.67	0.814
Fruits consumption ≥10.5 vs <10.5 portions/week	1.13	0.63	2.02	0.682

BMI: body mass index; GWG: gestational weight gain; CI: confidence interval; OR: Odds Ratio; LB: lower bound; UB: upper bound

Test: Logistic regression; $p \leq 0.05$ was considered significant

[a]Threshold of PFHpA was determined according to distribution

[b]Variables entered as predictors were age, parity, pre-pregnancy BMI, GWG, crowding index, education, having been breastfed, geographical vicinity to landfills, geographical vicinity to factories, illegal incinerations, smoking, passive smoking, alcohol consumption, fish / shellfish consumption, red meat consumption, poultry consumption, dairy products consumption, eggs consumption, and fruits consumption

[c] p-interaction (fish/shellfish consumption x education) = 0.468

[d] p-interaction (fish/shellfish consumption x vicinity to illegal incineration) = 0.346

Table S4. Logistic regression for maternal serum PFOA using backward method (N=269)

	95% CI			
	**OR[b]**	**LB**	**UB**	**p- value**
**PFOA High vs Low[a]**				
Fish / Shellfish consumption ≥0.63 vs <0.63 portion/week[c,d]	2.05	1.13	3.72	0.019
Vicinity to illegal incinerations Yes vs No[d]	1.92	1.02	3.60	0.043

Education University level vs High school or less[c]	2.22	1.18	4.18	0.014
Age ≥30 vs <30	0.83	0.44	1.54	0.552
Multiparous vs Primiparous	0.71	0.37	1.37	0.310
Pre-pregnancy BMI ≥25 vs <25 kg/m^2	0.91	0.48	1.73	0.770
GWG				
Normal vs Inadequate	1.12	0.55	2.29	0.752
Excessive vs Inadequate	1.04	0.49	2.21	0.917
Crowding index ≥1 vs <1	0.92	0.42	1.99	0.823
Breastfed Yes vs No	1.05	0.39	2.80	0.921
Vicinity to factories Yes vs No	0.70	0.39	1.26	0.237
Vicinity to landfills Yes vs No	1.13	0.59	2.13	0.718
Ever smoking Yes vs No	0.71	0.39	1.30	0.262
Passive smoking Yes vs No	1.44	0.71	2.90	0.315
Alcohol Yes vs No	0.42	0.18	1.01	0.052
Red meat consumption ≥ 1.94 vs <1.94 portions/week	1.59	0.90	2.81	0.111
Dairy products consumption ≥11.5 vs <11.5 portions/week	0.76	0.43	1.37	0.365
Poultry consumption ≥ 2 vs <2 portions/week	1.23	0.70	2.19	0.473
Eggs consumption ≥1.4 vs <1.4 portions/week	0.95	0.53	1.71	0.874
Fruits consumption ≥10.5 vs <10.5 portions/week	1.16	0.65	2.09	0.610

BMI: body mass index; GWG: gestational weight gain; CI: confidence interval; OR: Odds Ratio; LB: lower bound; UB: upper bound

Test: Logistic regression; $p \leq 0.05$ was considered significant

[a]Threshold of PFOA was determined according to distribution

[b]Variables entered as predictors were age, parity, pre-pregnancy BMI, GWG, crowding index, education, having been breastfed, geographical vicinity to landfills, geographical vicinity to factories, illegal incinerations, smoking, passive smoking, alcohol consumption, fish / shellfish consumption, red meat consumption, poultry consumption, dairy products consumption, eggs consumption, and fruits consumption

[c] p-interaction (fish/shellfish consumption x education) = 0.946

[d] p-interaction (fish/shellfish consumption x vicinity to illegal incineration) = 0.593

Table S5. Logistic regression for maternal serum PFHxS using backward method (N=269)

		95% CI		
	OR[b]	LB	UB	p- value
**PFHxS High vs Low[a]**				
Fish / Shellfish consumption ≥0.63 vs <0.63 portion/week[c,d]	1.84	1.02	3.31	0.042
Vicinity to illegal incinerations Yes vs No[d]	1.87	1.00	3.46	0.048
Education University level vs High school or less[c]	2.13	1.14	3.98	0.017
Age ≥30 vs <30	0.84	0.46	1.55	0.583
Multiparous vs Primiparous	0.75	0.39	1.44	0.390
Pre-pregnancy BMI ≥25 vs <25 kg/m^2	1.08	0.58	2.04	0.803
GWG				
Normal vs Inadequate	1.12	0.56	2.26	0.748
Excessive vs Inadequate	0.94	0.45	1.97	0.868

Crowding index ≥1 vs <1	0.98	0.46	2.09	0.955
Breastfed Yes vs No	0.93	0.35	2.43	0.880
Vicinity to factories Yes vs No	0.71	0.40	1.27	0.248
Vicinity to landfills Yes vs No	1.00	0.53	1.88	0.998
Ever smoking Yes vs No	0.84	0.46	1.52	0.558
Passive smoking Yes vs No	1.46	0.73	2.94	0.285
Alcohol Yes vs No	0.47	0.20	1.10	0.081
Red meat consumption ≥ 1.94 vs <1.94 portions/week	1.61	0.92	2.83	0.094
Dairy products consumption ≥11.5 vs <11.5 portions/week	0.72	0.40	1.27	0.256
Poultry consumption ≥ 2 vs <2 portions/week	1.43	0.81	2.51	0.217
Eggs consumption ≥1.4 vs <1.4 portions/week	0.80	0.45	1.43	0.460
Fruits consumption ≥10.5 vs <10.5 portions/week	1.18	0.66	2.10	0.575

BMI: body mass index; GWG: gestational weight gain; CI: confidence interval; OR: Odds Ratio; LB: lower bound; UB: upper bound

Test: Logistic regression; $p \leq 0.05$ was considered significant

[a]Threshold of PFHxS was determined according to distribution

[b]Variables entered as predictors were age, parity, pre-pregnancy BMI, GWG, crowding index, education, having been breastfed, geographical vicinity to landfills, geographical vicinity to factories, illegal incinerations, smoking, passive smoking, alcohol consumption, fish / shellfish consumption, red meat consumption, poultry consumption, dairy products consumption, eggs consumption, and fruits consumption

[c] p-interaction (fish/shellfish consumption x education) = 0.925

[d] p-interaction (fish/shellfish consumption x vicinity to illegal incineration) = 0.576

Table S6. Logistic regression for maternal serum PFOS using backward method (N=269)

		95% CI		
	**OR[b]**	**LB**	**UB**	**p- value**
**PFOS High vs Low[a]**				
Fish / Shellfish consumption ≥0.63 vs <0.63 portion/week[c,d]	1.90	1.05	3.46	0.034
Vicinity to illegal incinerations Yes vs No[d]	2.03	1.08	3.81	0.029
Education University level vs High school or less[c]	2.07	1.10	3.89	0.025
Age ≥30 vs <30	0.85	0.46	1.58	0.613
Multiparous vs Primiparous	0.70	0.36	1.36	0.293
Pre-pregnancy BMI ≥25 vs <25 kg/m^2	0.98	0.51	1.86	0.944
GWG				
Normal vs Inadequate	1.19	0.59	2.42	0.630
Excessive vs Inadequate	1.01	0.48	2.14	0.983
Crowding index ≥1 vs <1	0.96	0.45	2.09	0.927
Breastfed Yes vs No	1.02	0.38	2.70	0.971
Vicinity to factories Yes vs No	0.71	0.39	1.28	0.256
Vicinity to landfills Yes vs No	1.00	0.53	1.90	0.994
Ever smoking Yes vs No	0.72	0.40	1.32	0.292
Passive smoking Yes vs No	1.51	0.74	3.06	0.255
Alcohol Yes vs No	0.44	0.19	1.06	0.067
Red meat consumption ≥ 1.94 vs <1.94 portions/week	1.60	0.91	2.83	0.105

Dairy products consumption ≥11.5 vs <11.5 portions/week	0.72	0.40	1.29	0.274
Poultry consumption ≥ 2 vs <2 portions/week	1.26	0.71	2.24	0.422
Eggs consumption ≥1.4 vs <1.4 portions/week	0.99	0.55	1.78	0.977
Fruits consumption ≥10.5 vs <10.5 portions/week	1.10	0.61	1.97	0.746

BMI: body mass index; GWG: gestational weight gain; CI: confidence interval; OR: Odds Ratio; LB: lower bound; UB: upper bound

Test: Logistic regression; $p \leq 0.05$ was considered significant

[a]Threshold of PFOS was determined according to distribution

[b]Variables entered as predictors were age, parity, pre-pregnancy BMI, GWG, crowding index, education, having been breastfed, geographical vicinity to landfills, geographical vicinity to factories, illegal incinerations, smoking, passive smoking, alcohol consumption, fish / shellfish consumption, red meat consumption, poultry consumption, dairy products consumption, eggs consumption, and fruits consumption

[c] p-interaction (fish/shellfish consumption x education) = 0.984

[d] p-interaction (fish/shellfish consumption x vicinity to illegal incineration) = 0.569

Table S7. Association between dietary intake, use of tap water, and PFAS human milk levels (n=35)

		PFAS Categories				
		**Low (n=18)** N; %		**High (n=21)** N; %		***p*-Value[a]**
**∑PFAS**						
Dairy Products	<1.5portions/day	14	77.8%	8	38.1%	0.013
	≥1.5portions/day	4	22.2%	13	61.9%	
Eggs[b]	<1.68 portion/week (Median)	13	72.2%	6	28.6%	0.007
	>1.68 portion/week (Median)	5	27.8%	15	71.4%	
Use of Tap Water	Yes	0	0.0%	5	27.8%	0.019
	No	17	100.0%	13	72.2%	
**PFHpA**						
Dairy Products	<1.5portions/day	14	77.8%	8	38.1%	0.013
	≥1.5portions/day	4	22.2%	13	61.9%	
Eggs[b]	<1.68 portion/week (Median)	13	72.2%	6	28.6%	0.007
	>1.68 portion/week (Median)	5	27.8%	15	71.4%	
Use of Tap Water	Yes	0	0.0%	5	27.8%	0.019
	No	17	100.0%	13	72.2%	
**PFOA**						
Dairy Products	<1.5portions/day	14	77.8%	8	38.1%	0.013
	≥1.5portions/day	4	22.2%	13	61.9%	
Eggs[b]	<1.68 portion/week (Median)	13	72.2%	6	28.6%	0.007
	>1.68 portion/week (Median)	5	27.8%	15	71.4%	
Tap Water	Yes	0	0.0%	5	27.8%	0.019
	No	17	100.0%	13	72.2%	
**PFNA**						
Dairy Products	<1.5portions/day	14	77.8%	8	38.1%	0.013
	≥1.5portions/day	4	22.2%	13	61.9%	

Eggs[b]	<1.68 portion/week (Median)	13	72.2%	6	28.6%	0.007
	>1.68 portion/week (Median)	5	27.8%	15	71.4%	
Tap Water	Yes	0	0.0%	5	27.8%	0.019
	No	17	100.0%	13	72.2%	
**PFHxS**						
Dairy Products	<1.5portions/day	14	77.8%	8	38.1%	0.013
	≥1.5portions/day	4	22.2%	13	61.9%	
Eggs[b]	<1.68 portion/week (Median)	13	72.2%	6	28.6%	0.007
	>1.68 portion/week (Median)	5	27.8%	15	71.4%	
Tap Water	Yes	0	0.0%	5	27.8%	0.019
	No	17	100.0%	13	72.2%	
**PFOS**						
Dairy Products	<1.5portions/day	14	77.8%	8	38.1%	0.013
	≥1.5portions/day	4	22.2%	13	61.9%	
Eggs[b]	<1.68 portion/week (Median)	13	72.2%	6	28.6%	0.007
	>1.68 portion/week (Median)	5	27.8%	15	71.4%	
Tap Water	Yes	0	0.0%	5	27.8%	0.019
	No	17	100.0%	13	72.2%	

[a] statistical test: Chi-square
[b] significant in non-parametric test, p for all PFAS is 0.013
$p \leq 0.05$ was considered significant

Table S8. Multivariate Linear Regression for Z-scores of newborns weight-for-length at birth with maternal serum ∑PFAS as predictor (n=243)

		95% CI		
	**Unst. β[b]**	**LB**	**UB**	***p*-value**
**Weight-for-length**				
∑PFAS High vs Low[a]	-0.034	-0.069	0.001	0.057
Age ≥30 vs <30 years	-0.008	-0.043	0.027	0.656
Multiparous vs Primiparous	0.015	-0.024	0.054	0.459
Pre-pregnancy BMI ≥25 vs <25 kg/m^2	-0.030	-0.067	0.008	0.125
GWG	0.011	-0.011	0.032	0.325
Pregnancy weight loss from restrictive diet Yes vs No	0.028	-0.050	0.107	0.480
Pre-pregnancy weight loss from restrictive diet Yes vs No	-0.007	-0.051	0.036	0.739
Passive smoking Yes vs No	0.0004	-0.041	0.042	0.985
Smoking Yes vs Non	0.006	-0.029	0.041	0.735
Gestational age (In weeks)	0.010	-0.003	0.023	0.125
Crowding index >1 vs ≤1	-0.035	-0.080	0.011	0.132
Education University vs High school or less	0.038	0.001	0.075	0.042

BMI : Body mass index; Unst. β: Unstandardized Beta; CI: Confidence Interval; LB: Lower Bound; UB: Upper Bound; GWG: Gestational Weight Gain

Test: Linear Regression; $p \leq 0.05$ was considered significant

[a]Threshold was determined according to distribution

[b]Variables included as predictors were age, parity, Pre-pregnancy BMI, GWG, Pre-pregnancy and Pregnancy weight loss from restrictive diet, crowding index, education, smoking, and passive smoking

Table S9. Multivariate Linear Regression for Z-scores of newborns weight-for-length at birth with maternal serum PFOA as predictor (n=243)

		95% CI		
	**Unst. β[b]**	**LB**	**UB**	***p*-value**
**Weight-for-length**				
PFOA High vs Low[a]	-0.033	-0.068	0.002	0.065
Age ≥30 vs <30 years	-0.008	-0.043	0.027	0.656
Multiparous vs Primiparous	0.015	-0.025	0.054	0.463
Pre-pregnancy BMI ≥25 vs <25 kg/m^2	-0.030	-0.068	0.008	0.119
GWG	0.011	-0.011	0.033	0.323
Pregnancy weight loss from restrictive diet Yes vs No	0.028	-0.050	0.107	0.481
Pre-pregnancy weight loss from restrictive diet Yes vs No	-0.007	-0.050	0.037	0.763
Passive smoking Yes vs No	0.000	-0.041	0.041	0.995

Smoking Yes vs Non	0.006	-0.030	0.041	0.756
Gestational age (In weeks)	0.010	-0.003	0.023	0.135
Crowding index >1 vs ≤1	-0.035	-0.080	0.010	0.130
Education University vs High school or less	0.039	0.002	0.076	0.041

BMI : Body mass index; Unst. β: Unstandardized Beta; CI: Confidence Interval; LB: Lower Bound; UB: Upper Bound; GWG: Gestational Weight Gain

Test: Linear Regression; $p \leq 0.05$ was considered significant

[a]Threshold was determined according to distribution

[b]Variables included as predictors were age, parity, Pre-pregnancy BMI, GWG, Pre-pregnancy and Pregnancy weight loss from restrictive diet, crowding index, education, smoking, and passive smoking

Table S10. Multivariate Linear Regression for Z-scores of newborns weight-for-length at birth with maternal serum PFHxS as predictor (n=243)

		95% CI		
	**Unst. β[b]**	**LB**	**UB**	***p*-value**
**Weight-for-length**				
PFHxS High vs Low[a]	-0.032	-0.067	0.003	0.071
Age ≥30 vs <30 years	-0.008	-0.043	0.027	0.653
Multiparous vs Primiparous	0.015	-0.024	0.054	0.450
Pre-pregnancy BMI ≥25 vs <25 kg/m^2	-0.029	-0.067	0.009	0.128
GWG	0.011	-0.011	0.033	0.319
Pregnancy weight loss from restrictive diet Yes vs No	0.028	-0.050	0.107	0.478
Pre-pregnancy weight loss from restrictive diet Yes vs No	-0.008	-0.051	0.036	0.731
Passive smoking Yes vs No	0.00003	-0.041	0.041	0.999
Smoking Yes vs Non	0.007	-0.028	0.042	0.700
Gestational age (In weeks)	0.010	-0.003	0.023	0.128
Crowding index >1 vs ≤1	-0.034	-0.079	0.011	0.137
Education University vs High school or less	0.039	0.002	0.076	0.041

BMI : Body mass index; Unst. β: Unstandardized Beta; CI: Confidence Interval; LB: Lower Bound; UB: Upper Bound; GWG: Gestational Weight Gain

Test: Linear Regression; $p \leq 0.05$ was considered significant

[a]Threshold was determined according to distribution

[b]Variables included as predictors were age, parity, Pre-pregnancy BMI, GWG, Pre-pregnancy and Pregnancy weight loss from restrictive diet, crowding index, education, smoking, and passive smoking

Table S11. Multivariate Linear Regression for Z-scores of newborns weight-for-length at birth with maternal serum PFOS as predictor (n=243)

		95% CI		
	**Unst. β[b]**	**LB**	**UB**	***p*-value**
**Weight-for-length**				
PFOS High vs Low[a]	-0.034	-0.069	0.001	0.057
Age ≥30 vs <30 years	-0.008	-0.043	0.027	0.656
Multiparous vs Primiparous	0.015	-0.024	0.054	0.459
Pre-pregnancy BMI ≥25 vs <25 kg/m^2	-0.030	-0.067	0.008	0.125
GWG	0.011	-0.011	0.032	0.325
Pregnancy weight loss from restrictive diet Yes vs No	0.028	-0.050	0.107	0.480
Pre-pregnancy weight loss from restrictive diet Yes vs No	-0.007	-0.051	0.036	0.739
Passive smoking Yes vs No	0.000	-0.041	0.042	0.985
Smoking Yes vs Non	0.006	-0.029	0.041	0.735
Gestational age (In weeks)	0.010	-0.003	0.023	0.125
Crowding index >1 vs ≤1	-0.035	-0.080	0.011	0.132
Education University vs High school or less	0.038	0.001	0.075	0.042

BMI : Body mass index; Unst. β: Unstandardized Beta; CI: Confidence Interval; LB: Lower Bound; UB: Upper Bound; GWG: Gestational Weight Gain

Test: Linear Regression; $p \leq 0.05$ was considered significant

[a]Threshold was determined according to distribution

[b]Variables included as predictors were age, parity, Pre-pregnancy BMI, GWG, Pre-pregnancy and Pregnancy weight loss from restrictive diet, crowding index, education, smoking, and passive smoking

Table S12. Multivariate Linear Regression for Z scores of newborns anthropometric measurements at birth with PFHpA as predictor (n=243)

			CI 95%		
	Unst. β	St. β	LB	UB	*p*-value
**Weight-for-age**					
PFHpA High vs Low[a]	-0.007	-0.026	-0.042	0.027	0.677
Age ≥30 vs <30 years	0.016	0.059	-0.019	0.051	0.367
Multiparous vs Primiparous	0.015	0.054	-0.024	0.054	0.449
Pre-pregnancy BMI ≥25 vs <25 kg/m^2	-0.006	-0.023	-0.043	0.031	0.737
GWG[c]	0.022	0.135	0.001	0.043	0.043
Pregnancy weight loss from restrictive diet Yes vs No	0.032	0.054	-0.045	0.109	0.411
Pre-pregnancy weight loss from restrictive diet Yes vs No	0.001	0.003	-0.042	0.044	0.959
Passive smoking Yes vs No	0.002	0.008	-0.038	0.043	0.905
Smoking Yes vs Non	-0.012	-0.044	-0.047	0.023	0.506
Gestational age (In weeks)	0.022	0.216	0.009	0.035	0.001
Crowding index >1 vs ≤1	-0.021	-0.062	-0.065	0.023	0.344
Education University vs High school or less	0.021	0.078	-0.015	0.058	0.250
**Length-for-age**					
PFHpA High vs Low[a]	-0.009	-0.051	-0.032	0.015	0.467
Age ≥30 vs <30 years	0.016	0.100	-0.007	0.040	0.177
Multiparous vs Primiparous	0.011	0.067	-0.015	0.037	0.398
Pre-pregnancy BMI ≥25 vs <25 kg/m^2	-0.005	-0.027	-0.030	0.021	0.726
GWG[c]	-0.002	-0.020	-0.017	0.013	0.788
Pregnancy weight loss from restrictive diet Yes vs No	-0.009	-0.026	-0.060	0.042	0.730
Pre-pregnancy weight loss from restrictive diet Yes vs No	0.016	0.082	-0.013	0.046	0.279
Passive smoking Yes vs No	0.020	0.100	-0.008	0.047	0.155
Smoking Yes vs Non	0.001	0.006	-0.022	0.024	0.938
Gestational age (In weeks)	0.001	0.011	-0.010	0.011	0.876
Crowding index >1 vs ≤1	-0.002	-0.007	-0.032	0.029	0.918
Education University vs High school or less	-0.003	-0.015	-0.027	0.022	0.834
**Head circumference-for-age**					
PFHpA High vs Low[a]	-0.0005	-0.005	-0.014	0.013	0.944
Age ≥30 vs <30 years	0.009	0.095	-0.005	0.022	0.196
Multiparous vs Primiparous	0.022	0.231	0.007	0.037	0.004

Pre-pregnancy BMI ≥25 vs <25 kg/m^2	0.008	0.084	-0.007	0.023	0.278
GWG[c]	-0.002	-0.034	-0.010	0.007	0.657
Pregnancy weight loss from restrictive diet Yes vs No	0.008	0.044	-0.020	0.037	0.558
Pre-pregnancy weight loss from restrictive diet Yes vs No	-0.009	-0.080	-0.026	0.008	0.298
Passive smoking Yes vs No	0.009	0.078	-0.007	0.024	0.263
Smoking Yes vs Non	-0.004	-0.044	-0.017	0.009	0.529
Gestational age (In weeks)	0.009	0.215	0.003	0.015	0.004
Crowding index >1 vs ≤1	-0.015	-0.127	-0.032	0.002	0.081
Education University vs High school or less	0.013	0.136	-0.001	0.027	0.066

BMI : Body mass index; Unst. β: Unstandardized Beta; St. β: Standardized Beta; CI: Confidence Interval; LB: Lower Bound; UB: Upper Bound; GWG: Gestational Weight Gain

Test: Linear Regression; $p \leq 0.05$ was considered significant

[a]Cut-offs of PFHpA were determined according to distribution

[b]Variables included as predictors were age, parity, Pre-pregnancy BMI, GWG, Pre-pregnancy and Pregnancy weight loss from restrictive diet, crowding index, education, smoking, and passive smoking

[c]GWG was categorized according to Institute of Medicine recommendations (IOM) (IOM and NRC, 2009)

Table S13. Multivariate Linear Regression for Z scores of newborns anthropometric measurements at birth with PFOA as predictor (n=243)

			CI 95%		
	**Unst. β**	**St. β**	**LB**	**UB**	***p*-value**
**Weight-for-age**					
PFOA High vs Low[a]	-0.010	-0.038	-0.045	0.024	0.553
Age ≥30 vs <30 years	0.016	0.058	-0.019	0.050	0.374
Multiparous vs Primiparous	0.015	0.053	-0.024	0.054	0.456
Pre-pregnancy BMI ≥25 vs <25 kg/m^2	-0.006	-0.023	-0.043	0.031	0.732
GWG[c]	0.022	0.136	0.001	0.043	0.042
Pregnancy weight loss from restrictive diet Yes vs No	0.031	0.052	-0.047	0.108	0.434
Pre-pregnancy weight loss from restrictive diet Yes vs No	0.002	0.005	-0.041	0.044	0.944
Passive smoking Yes vs No	0.002	0.008	-0.038	0.043	0.906
Smoking Yes vs Non	-0.012	-0.044	-0.047	0.023	0.502
Gestational age (In weeks)	0.022	0.216	0.009	0.035	0.001
Crowding index >1 vs ≤1	-0.021	-0.063	-0.065	0.023	0.340
Education University vs High school or less	0.022	0.080	-0.015	0.058	0.239
**Length-for-age**					
PFOA High vs Low[a]	-0.010	-0.059	-0.033	0.013	0.402
Age ≥30 vs <30 years	0.016	0.098	-0.008	0.040	0.184
Multiparous vs Primiparous	0.011	0.066	-0.015	0.037	0.405
Pre-pregnancy BMI ≥25 vs <25 kg/m^2	-0.005	-0.027	-0.030	0.021	0.721
GWG[c]	-0.002	-0.019	-0.016	0.013	0.806
Pregnancy weight loss from restrictive diet Yes vs No	-0.010	-0.027	-0.061	0.041	0.712
Pre-pregnancy weight loss from restrictive diet Yes vs No	0.016	0.083	-0.013	0.046	0.275
Passive smoking Yes vs No	0.020	0.100	-0.007	0.047	0.154
Smoking Yes vs Non	0.001	0.005	-0.022	0.024	0.942
Gestational age (In weeks)	0.001	0.010	-0.010	0.011	0.893
Crowding index >1 vs ≤1	-0.002	-0.008	-0.032	0.029	0.915
Education University vs High school or less	-0.002	-0.014	-0.027	0.022	0.846
**Head circumference-for-age**					
PFOA High vs Low[a]	-0.003	-0.031	-0.016	0.010	0.659
Age ≥30 vs <30 years	0.009	0.095	-0.005	0.022	0.197
Multiparous vs Primiparous	0.022	0.230	0.007	0.037	0.004

Pre-pregnancy BMI ≥25 vs <25 kg/m^2	0.008	0.083	-0.007	0.023	0.280
GWG[c]	-0.002	-0.033	-0.010	0.007	0.662
Pregnancy weight loss from restrictive diet Yes vs No	0.007	0.038	-0.021	0.035	0.616
Pre-pregnancy weight loss from restrictive diet Yes vs No	-0.009	-0.078	-0.026	0.008	0.310
Passive smoking Yes vs No	0.009	0.080	-0.006	0.024	0.255
Smoking Yes vs Non	-0.004	-0.045	-0.017	0.009	0.524
Gestational age (In weeks)	0.009	0.213	0.003	0.015	0.004
Crowding index >1 vs ≤1	-0.015	-0.129	-0.032	0.002	0.075
Education University vs High school or less	0.013	0.140	0.000	0.027	0.058

BMI : Body mass index; Unst. β: Unstandardized Beta; St. β: Standardized Beta; CI: Confidence Interval; LB: Lower Bound; UB: Upper Bound; GWG: Gestational Weight Gain

Test: Linear Regression; $p \leq 0.05$ was considered significant

[a]Cut-offs of PFOA were determined according to distribution

[b]Variables included as predictors were age, parity, Pre-pregnancy BMI, GWG, Pre-pregnancy and Pregnancy weight loss from restrictive diet, crowding index, education, smoking, and passive smoking

[c]GWG was categorized according to Institute of Medicine recommendations (IOM) (IOM and NRC, 2009)

Table S14. Multivariate Linear Regression for Z scores of newborns anthropometric measurements at birth with PFHxS as predictor (n=243)

			CI 95%		
	**Unst. β**	**St. β**	**LB**	**UB**	***p*-value**
**Weight-for-age**					
PFHxS High vs Low[a]	-0.010	-0.035	-0.044	0.025	0.584
Age ≥30 vs <30 years	0.016	0.059	-0.019	0.050	0.372
Multiparous vs Primiparous	0.015	0.054	-0.024	0.054	0.451
Pre-pregnancy BMI ≥25 vs <25 kg/m^2	-0.006	-0.022	-0.043	0.031	0.745
GWG[c]	0.022	0.135	0.001	0.043	0.043
Pregnancy weight loss from restrictive diet Yes vs No	0.031	0.052	-0.047	0.108	0.433
Pre-pregnancy weight loss from restrictive diet Yes vs No	0.001	0.004	-0.041	0.044	0.947
Passive smoking Yes vs No	0.002	0.008	-0.038	0.043	0.907
Smoking Yes vs Non	-0.011	-0.042	-0.046	0.023	0.517
Gestational age (In weeks)	0.022	0.217	0.009	0.036	0.001
Crowding index >1 vs ≤1	-0.021	-0.063	-0.065	0.023	0.341
Education University vs High school or less	0.022	0.079	-0.015	0.058	0.243
**Length-for-age**					
PFHxS High vs Low[a]	-0.009	-0.053	-0.032	0.014	0.453
Age ≥30 vs <30 years	0.016	0.099	-0.007	0.040	0.179
Multiparous vs Primiparous	0.011	0.067	-0.015	0.037	0.400
Pre-pregnancy BMI ≥25 vs <25 kg/m^2	-0.004	-0.026	-0.030	0.021	0.735
GWG[c]	-0.002	-0.020	-0.017	0.013	0.797
Pregnancy weight loss from restrictive diet Yes vs No	-0.009	-0.027	-0.060	0.042	0.721
Pre-pregnancy weight loss from restrictive diet Yes vs No	0.016	0.083	-0.013	0.046	0.276
Passive smoking Yes vs No	0.020	0.099	-0.008	0.047	0.158
Smoking Yes vs Non	0.001	0.007	-0.022	0.024	0.924
Gestational age (In weeks)	0.001	0.011	-0.010	0.011	0.884
Crowding index >1 vs ≤1	-0.002	-0.008	-0.032	0.029	0.913
Education University vs High school or less	-0.002	-0.015	-0.027	0.022	0.843
**Head circumference-for-age**					
PFHxS High vs Low[a]	-0.002	-0.024	-0.015	0.011	0.739
Age ≥30 vs <30 years	0.009	0.095	-0.005	0.022	0.196
Multiparous vs Primiparous	0.022	0.230	0.007	0.037	0.004

Pre-pregnancy BMI ≥25 vs <25 kg/m^2	0.008	0.084	-0.007	0.023	0.276
GWG[c]	-0.002	-0.034	-0.010	0.007	0.658
Pregnancy weight loss from restrictive diet Yes vs No	0.007	0.039	-0.021	0.036	0.604
Pre-pregnancy weight loss from restrictive diet Yes vs No	-0.009	-0.078	-0.026	0.008	0.308
Passive smoking Yes vs No	0.009	0.079	-0.007	0.024	0.259
Smoking Yes vs Non	-0.004	-0.044	-0.017	0.009	0.532
Gestational age (In weeks)	0.009	0.214	0.003	0.015	0.004
Crowding index >1 vs ≤1	-0.015	-0.129	-0.032	0.002	0.076
Education University vs High school or less	0.013	0.139	-0.001	0.027	0.060

BMI : Body mass index; Unst. β: Unstandardized Beta; St. β: Standardized Beta; CI: Confidence Interval; LB: Lower Bound; UB: Upper Bound; GWG: Gestational Weight Gain

Test: Linear Regression; $p \leq 0.05$ was considered significant

[a]Cut-offs of PFHxS were determined according to distribution

[b]Variables included as predictors were age, parity, Pre-pregnancy BMI, GWG, Pre-pregnancy and Pregnancy weight loss from restrictive diet, crowding index, education, smoking, and passive smoking

[c]GWG was categorized according to Institute of Medicine recommendations (IOM) (IOM and NRC, 2009)

Table S15. Multivariate Linear Regression for Z scores of newborns anthropometric measurements at birth with PFOS as predictor (n=243)

			CI 95%		
	**Unst. β**	**St. β**	**LB**	**UB**	***p*-value**
**Weight-for-age**					
PFOS High vs Low[a]	-0.009	-0.032	-0.044	0.026	0.617
Age ≥30 vs <30 years	0.016	0.059	-0.019	0.050	0.371
Multiparous vs Primiparous	0.015	0.053	-0.024	0.054	0.453
Pre-pregnancy BMI ≥25 vs <25 kg/m^2	-0.006	-0.023	-0.043	0.031	0.738
GWG[c]	0.022	0.135	0.001	0.043	0.043
Pregnancy weight loss from restrictive diet Yes vs No	0.032	0.053	-0.046	0.109	0.421
Pre-pregnancy weight loss from restrictive diet Yes vs No	0.001	0.004	-0.041	0.044	0.955
Passive smoking Yes vs No	0.002	0.008	-0.038	0.043	0.906
Smoking Yes vs Non	-0.012	-0.043	-0.047	0.023	0.508
Gestational age (In weeks)	0.022	0.217	0.009	0.036	0.001
Crowding index >1 vs ≤1	-0.021	-0.062	-0.065	0.023	0.343
Education University vs High school or less	0.022	0.079	-0.015	0.058	0.246
**Length-for-age**					
PFOS High vs Low[a]	-0.009	-0.057	-0.033	0.014	0.421
Age ≥30 vs <30 years	0.016	0.099	-0.008	0.040	0.180
Multiparous vs Primiparous	0.011	0.067	-0.015	0.037	0.401
Pre-pregnancy BMI ≥25 vs <25 kg/m^2	-0.005	-0.027	-0.030	0.021	0.728
GWG[c]	-0.002	-0.020	-0.017	0.013	0.795
Pregnancy weight loss from restrictive diet Yes vs No	-0.009	-0.027	-0.060	0.042	0.717
Pre-pregnancy weight loss from restrictive diet Yes vs No	0.016	0.083	-0.013	0.046	0.275
Passive smoking Yes vs No	0.020	0.100	-0.008	0.047	0.156
Smoking Yes vs Non	0.001	0.006	-0.022	0.024	0.935
Gestational age (In weeks)	0.001	0.011	-0.010	0.011	0.878
Crowding index >1 vs ≤1	-0.002	-0.008	-0.032	0.029	0.917
Education University vs High school or less	-0.003	-0.015	-0.027	0.022	0.838
**Head circumference-for-age**					
PFOS High vs Low[a]	-0.002	-0.025	-0.016	0.011	0.723
Age ≥30 vs <30 years	0.009	0.095	-0.005	0.022	0.196
Multiparous vs Primiparous	0.022	0.230	0.007	0.037	0.004

Pre-pregnancy BMI ≥25 vs <25 kg/m^2	0.008	0.084	-0.007	0.023	0.278
GWG[c]	-0.002	-0.034	-0.010	0.007	0.657
Pregnancy weight loss from restrictive diet Yes vs No	0.007	0.039	-0.021	0.036	0.603
Pre-pregnancy weight loss from restrictive diet Yes vs No	-0.009	-0.078	-0.026	0.008	0.308
Passive smoking Yes vs No	0.009	0.079	-0.007	0.024	0.258
Smoking Yes vs Non	-0.004	-0.044	-0.017	0.009	0.527
Gestational age (In weeks)	0.009	0.214	0.003	0.015	0.004
Crowding index >1 vs ≤1	-0.015	-0.129	-0.032	0.002	0.076
Education University vs High school or less	0.013	0.138	-0.001	0.027	0.060

BMI : Body mass index; Unst. β: Unstandardized Beta; St. β: Standardized Beta; CI: Confidence Interval; LB: Lower Bound; UB: Upper Bound; GWG: Gestational Weight Gain

Test: Linear Regression; $p \leq 0.05$ was considered significant

[a]Cut-offs of PFOS were determined according to distribution

[b]Variables included as predictors were age, parity, Pre-pregnancy BMI, GWG, Pre-pregnancy and Pregnancy weight loss from restrictive diet, crowding index, education, smoking, and passive smoking

[c]GWG was categorized according to Institute of Medicine recommendations (IOM) (IOM and NRC, 2009)

Table S16. Multivariate Linear Regression for Z scores of newborns anthropometric measurements at birth with ∑PFAS as predictor (n=243)

			CI 95%		
	**Unst. β**	**St. β**	**LB**	**UB**	***p*-value**
**Weight-for-age**					
∑PFAS High vs Low[a]	-0.009	-0.032	-0.044	0.026	0.617
Age ≥30 vs <30 years	0.016	0.059	-0.019	0.050	0.371
Multiparous vs Primiparous	0.015	0.053	-0.024	0.054	0.453
Pre-pregnancy BMI ≥25 vs <25 kg/m^2	-0.006	-0.023	-0.043	0.031	0.738
GWG[c]	0.022	0.135	0.001	0.043	0.043
Pregnancy weight loss from restrictive diet Yes vs No	0.032	0.053	-0.046	0.109	0.421
Pre-pregnancy weight loss from restrictive diet Yes vs No	0.001	0.004	-0.041	0.044	0.955
Passive smoking Yes vs No	0.002	0.008	-0.038	0.043	0.906
Smoking Yes vs Non	-0.012	-0.043	-0.047	0.023	0.508
Gestational age (In weeks)	0.022	0.217	0.009	0.036	0.001
Crowding index >1 vs ≤1	-0.021	-0.062	-0.065	0.023	0.343
Education University vs High school or less	0.022	0.079	-0.015	0.058	0.246
**Length-for-age**					
∑PFAS High vs Low[a]	-0.009	-0.057	-0.033	0.014	0.421
Age ≥30 vs <30 years	0.016	0.099	-0.008	0.040	0.180
Multiparous vs Primiparous	0.011	0.067	-0.015	0.037	0.401
Pre-pregnancy BMI ≥25 vs <25 kg/m^2	-0.005	-0.027	-0.030	0.021	0.728
GWG[c]	-0.002	-0.020	-0.017	0.013	0.795
Pregnancy weight loss from restrictive diet Yes vs No	-0.009	-0.027	-0.060	0.042	0.717
Pre-pregnancy weight loss from restrictive diet Yes vs No	0.016	0.083	-0.013	0.046	0.275
Passive smoking Yes vs No	0.020	0.100	-0.008	0.047	0.156
Smoking Yes vs Non	0.001	0.006	-0.022	0.024	0.935
Gestational age (In weeks)	0.001	0.011	-0.010	0.011	0.878
Crowding index >1 vs ≤1	-0.002	-0.008	-0.032	0.029	0.917
Education University vs High school or less	-0.003	-0.015	-0.027	0.022	0.838
**Head circumference-for-age**					
∑PFAS High vs Low[a]	-0.002	-0.025	-0.016	0.011	0.723
Age ≥30 vs <30 years	0.009	0.095	-0.005	0.022	0.196
Multiparous vs Primiparous	0.022	0.230	0.007	0.037	0.004

Pre-pregnancy BMI ≥25 vs <25 kg/m^2	0.008	0.084	-0.007	0.023	0.278
GWG[c]	-0.002	-0.034	-0.010	0.007	0.657
Pregnancy weight loss from restrictive diet Yes vs No	0.007	0.039	-0.021	0.036	0.603
Pre-pregnancy weight loss from restrictive diet Yes vs No	-0.009	-0.078	-0.026	0.008	0.308
Passive smoking Yes vs No	0.009	0.079	-0.007	0.024	0.258
Smoking Yes vs Non	-0.004	-0.044	-0.017	0.009	0.527
Gestational age (In weeks)	0.009	0.214	0.003	0.015	0.004
Crowding index >1 vs ≤1	-0.015	-0.129	-0.032	0.002	0.076
Education University vs High school or less	0.013	0.138	-0.001	0.027	0.060

BMI : Body mass index; Unst. β: Unstandardized Beta; St. β: Standardized Beta; CI: Confidence Interval; LB: Lower Bound; UB: Upper Bound; GWG: Gestational Weight Gain

Test: Linear Regression; $p \leq 0.05$ was considered significant

[a]Cut-offs of ∑PFAS were determined according to distribution

[b]Variables included as predictors were age, parity, Pre-pregnancy BMI, GWG, Pre-pregnancy and Pregnancy weight loss from restrictive diet, crowding index, education, smoking, and passive smoking

[c]GWG was categorized according to Institute of Medicine recommendations (IOM) (IOM and NRC, 2009)
